# Evaluation of Secondhand Smoke Exposure in New York City Public Housing After Implementation of the 2018 Federal Smoke-Free Housing Policy

**DOI:** 10.1001/jamanetworkopen.2020.24385

**Published:** 2020-11-05

**Authors:** Lorna E. Thorpe, Elle Anastasiou, Katarzyna Wyka, Albert Tovar, Emily Gill, Ana Rule, Brian Elbel, Sue A. Kaplan, Nan Jiang, Terry Gordon, Donna Shelley

**Affiliations:** 1 Department of Population Health, NYU Grossman School of Medicine, New York; 2 Department of Epidemiology and Biostatistics, The City University of New York Graduate School of Public Health and Health Policy, New York; 3 Department of Environmental Health and Engineering, Johns Hopkins Bloomberg School of Public Health, Baltimore, Maryland; 4 Department of Environmental Medicine, NYU Grossman School of Medicine, New York; 5 Department of Public Health Policy and Management, NYU School of Global Public Health, New York

## Abstract

**Importance:**

Secondhand smoke (SHS) exposure is associated with many health conditions in children and adults. Millions of individuals in the US are currently exposed to SHS in their homes.

**Objective:**

To investigate whether a federal ban on smoking in public housing settings was associated with a decrease in indoor SHS levels in New York City public housing developments 12 months after the policy’s implementation.

**Design, Setting, and Participants:**

This cohort study tracked indoor air quality longitudinally from April 2018 to September 2019 and used difference-in-differences analysis to examine SHS exposure before vs after implementation of the 2018 federal smoke-free housing (SFH) policy in 10 New York City Housing Authority (NYCHA) buildings vs 11 matched low-income buildings not subject to the SFH policy (ie, Section 8 buildings).

**Exposures:**

Federal SFH policy implementation, beginning July 30, 2018.

**Main Outcomes and Measures:**

Comparison of nicotine concentration levels from passive, bisulfate-coated filters before vs 12 months after implementation of the federal SFH policy. Secondary outcomes included changes in particulate matter less than 2.5 μm in diameter, measured with low-cost particle monitors, and counts of cigarette butts in common areas.

**Results:**

Air quality was measured repeatedly in a total of 153 NYCHA and 110 Section 8 nonsmoking households as well as in 91 stairwells and hallways. Before the SFH policy implementation, air nicotine was detectable in 19 of 20 stairwells (95.0%) in NYCHA buildings and 15 of 19 stairwells (78.9%) in Section 8 buildings (*P* = .19) and in 17 of 19 hallways (89.5%) in NYCHA buildings and 14 of 23 hallways (60.9%) in Section 8 buildings (*P* = .004). Nicotine was detected less frequently inside nonsmoking apartments overall (26 of 263 [9.9%]) but more frequently in NYCHA apartments (20 of 153 [13.1%]) than in Section 8 apartments (6 of 110 [5.5%]) (*P* = .04). One year after policy implementation, there was no differential change over time in nicotine concentrations measured in stairwells (DID, 0.03 μg/m^3^; 95% CI, –0.99 to 1.06 μg/m^3^) or inside nonsmoking households (DID, –0.04 μg/m^3^; 95% CI, –0.24 to 0.15 μg/m^3^). Larger decreases in nicotine concentration were found in NYCHA hallways than in Section 8 hallways (DID, –0.43 μg/m^3^; 95% CI, –1.26 to 0.40 μg/m^3^).

**Conclusions and Relevance:**

The findings suggest that there was no differential change in SHS in NYCHA buildings 12 months after SFH policy implementation. Additional support may be needed to ensure adherence to SFH policies.

## Key Points

**Question:**

Was implementation of a federal smoke-free housing policy prohibiting indoor smoking in public housing associated with a decrease in secondhand smoke 12 months later?

**Findings:**

In this cohort study tracking indoor air quality inside the homes of 263 nonsmoking families and in common areas across 10 New York City public housing buildings vs 11 matched low-income buildings not subject to the smoke-free housing policy, no differential change in air nicotine or levels of particulate matter between housing types was found.

**Meaning:**

The findings suggest that additional support may be needed to ensure adherence to smoke-free housing policies and achieve desired public health outcomes.

## Teaser

This cohort study examines whether the 2018 implementation of a federal ban on smoking in public housing settings was associated with a decrease in indoor levels of secondhand smoke in New York City (NYC) public housing developments 12 months later.

## Introduction

Tobacco and involuntary exposure to secondhand smoke (SHS) remain leading causes of preventable death in the US, with more than 480 000 deaths annually.^1^ A large body of research has documented health hazards associated with SHS exposure. These include cancers, cardiovascular disease, and respiratory complications among adults and sudden infant death syndrome, respiratory infections, and asthma among children.^2,3^ Population-based studies using serologic cotinine biomarkers performed during the past 30 years have confirmed that exposure to SHS has decreased owing in great part to reductions in cigarette smoking and laws prohibiting smoking in public settings such as bars, restaurants, and workplaces. Among US residents who do not smoke, the prevalence of SHS exposure between 1988 and 2014 decreased from 87.5% to 25.2%.^4^

Despite this progress, reducing SHS exposure remains a public health priority. Nationally, approximately 1 in 4 individuals who do not smoke (58 million individuals) are still exposed to SHS routinely, primarily at home.^5^ Persistent disparities in SHS exposure are widely documented, with exposure being highest among racial/ethnic minority populations and persons with low income, reflecting higher smoking prevalence^6,7,8,9^ and differential risks across housing and social environments.^10,11,12,13^ Pursuant to a recent rule passed by the US Department of Housing and Urban Development (HUD), all public housing authorities were required to implement smoke-free housing (SFH) policies in their developments beginning July 30, 2018.^14^ Few studies have prospectively evaluated the association of SFH policies with SHS exposure using objectively measured airborne nicotine concentration or particulate matter less than 2.5 μm in diameter (PM_2.5_).^15,16,17,18,19,20^ These studies have had mixed results, mostly showing initial or no associations with SHS exposure, but follow-up duration and sample sizes have been limited.

As part of a larger natural experiment study to assess consequences of the SFH policy on health outcomes, we prospectively measured air quality before and 12 months after SFH policy implementation in 21 large public housing and private sector high-rise buildings housing predominantly low-income residents in New York City (NYC). This city is an important setting in which to examine consequences of the SFH policy because the NYC Housing Authority (NYCHA) is the largest housing authority in the US, with roughly 400 000 residents. Nearly 100 000 additional NYC households receive housing vouchers to subsidize apartment rental costs in private-sector buildings (herein referred to as *Section 8*). The goal of this analysis was to empirically assess the association of the SFH policy with SHS exposure in its first year of implementation using large samples of apartments and common areas.

## Methods

### Study Population

For this cohort study, we selected a purposeful sample of 21 high-rise buildings located in Manhattan and the Bronx, including 10 NYCHA buildings subject to the SFH policy and 11 Section 8 buildings. The following building eligibility criteria were established to ensure homogeneity: (1) buildings were high-rise (>15 floors); (2) buildings had a large resident population (>150 units); (3) at least 80% of the resident population were Black or Hispanic individuals (mirroring distribution in most NYCHA buildings); and (4) at least 20% of the resident population was younger than 18 years. The NYU Grossman School of Medicine institutional review board approved the study protocol, and all study participants signed a written informed consent form. This study followed the Transparent Reporting of Evaluations With Nonrandomized Designs (TREND) reporting guideline.^21^ Detailed information on the study population and procedures has been described elsewhere.^22^

### Study Design and Procedures

Using rosters of residents obtained from NYCHA and building managers, we conducted a baseline telephone survey to assess smoking prevalence and experience with SHS incursions.^23^ We conducted the survey among NYCHA residents and the subsequent prepolicy air-monitoring data collection from April to July 2018, immediately before the federal SFH rule went into effect, and in the 11 selected Section 8 buildings immediately thereafter, from August to November 2018, before the onset of cold weather. We targeted households that were above the third floor and had working telephone numbers to avoid biasing influences of ambient outdoor PM_2.5_. Adults aged 18 years or older who spoke English or Spanish were eligible for participation; we invited 1 member per household to participate in a 15-minute telephone survey that included questions about demographic characteristics, cigarette smoking behavior, and SHS incursions. We conducted door-to-door enrollment in person using tablets among households not reached by telephone. The survey yielded 1030 responses (559 NYCHA residents and 471 Section 8 residents), for a response rate of 35.3% among NYCHA residents and 32.1% among Section 8 residents.

At the conclusion of the survey, we invited a volunteer sample of 275 nonsmoking households (157 NYCHA households and 118 Section 8 households) to enroll in a longitudinal air-monitoring study to track air quality in 7-day stretches inside their homes every 6 months for 2.5 years (5 waves in total). This sample size was selected a priori based on statistical power considerations detailed in Cardozo et al.^22^ We excluded 12 households from this analysis based on evidence of probable indoor smoking by residents or guests (8 households) or incomplete air quality data at baseline (4 households), for an analytic sample size of 263 nonsmoking households (153 in NYCHA buildings and 110 in Section 8 buildings) (eFigure in the Supplement). A mean of 16 (range, 14-19) households per NYCHA building and 11 (range, 5-18) households per Section 8 building participated.

After conducting air-monitoring sessions among the 263 nonsmoking households before implementation of the SFH policy, we repeated air monitoring 6 months (December 2018 to March 2019) and 12 months (May 2019 to September 2019) after policy implementation. We compared findings before vs 12 months after policy implementation using comparable summer months to avoid seasonal influences. Of the 263 households enrolled, we completed air monitoring 12 months after the policy in 230 households, for a 1-year response rate of 87.5%. In addition to nonsmoking households, we also monitored air quality in 2 randomly selected hallways and 2 randomly selected stairwells per building for 7-day stretches in each wave (45 measurements in NYCHA buildings and 46 in Section 8 buildings).

### Objective Air Quality and SHS Measures

Experts have emphasized the superiority of air-quality monitoring over biological cotinine to measure SHS exposure in housing.^17,24,25,26^ Therefore, the primary measure of SHS incursions in this study was airborne nicotine concentration measured over a 7-day period using passive, bisulfate-coated filters placed in living rooms of enrolled nonsmoking households and common areas. Airborne nicotine concentration has been widely recognized in studies of SHS as a highly specific marker of tobacco smoking.^11,16,27,28^ Passive samplers were prepared and analyzed at Johns Hopkins University Bloomberg School of Public Health using the school’s Secondhand Smoke Exposure Assessment Laboratory standard operating procedures.^29^ The nicotine detection limit for 7-day samples was 0.017 μg/m^3^. We also measured ambient PM_2.5_ inside homes (secondary outcome) using AirBeam monitors (HabitatMap), which are novel low-cost particle sensors,^30^ and counted cigarette butts in common areas twice per wave in 4 randomly selected hallways and 2 randomly selected stairwells in the bottom 10 floors per building, averaging the numbers of cigarette butts counted. Detailed information on the study methods is described elsewhere.^22^

### Statistical Analysis

We tabulated demographic variables, smoking prevalence, and prevalence of SHS incursions from baseline survey responses and compared differences among NYCHA and Section 8 residents using χ^2^ statistics. For air nicotine concentration, we calculated geometric means and estimated the percentage of filters with detectable nicotine.^22^ Estimates were computed separately for common areas (stairwells and hallways) and nonsmoking apartments. For PM_2.5_, we calculated means and the percentage of readings with levels greater than the Environmental Protection Agency annual standard^31^ of 12.0 μg/m^3^. For cigarette butt counts, we calculated means separately for stairwells and hallways.

We used a difference-in-difference (DID) approach to compare within-group changes in air quality between NYCHA vs Section 8 buildings before vs 12 months after SFH policy implementation using mixed linear regression models for the 2 air quality measures (nicotine concentration and PM_2.5_ concentration) in stairwells and hallways as well as for cigarette butt counts in stairwells and hallways. We modeled outcomes for nicotine concentration by taking the natural log of the original nicotine concentration to account for skewedness. All regression models included fixed effects for study arm (NYCHA vs Section 8), data collection wave (before vs 12 months after policy implementation), and their interaction and adjusted for clustering of units nested within buildings and repeated measurements over time. Model-based mean differences with 95% CIs before SFH policy implementation to 12 months after SFH policy implementation were calculated for each outcome measure. The DID estimates compared the magnitude of differential changes in NYCHA vs Section 8 buildings. Exact *P* values were reported; the significance level was set at *P* < .05 using a 2-sided test. To account for the possible influence of systematic variation in outdoor air quality between groups, we repeated the DID model adjusting for outdoor ambient PM_2.5_ levels. Because ambient air pollution varies spatially more than temporally across NYC over short durations, we opted to use spatially granular zip code data from the 2017 NYC Community Air Survey.^32^ All analyses were performed using SAS statistical software, version 9.4 (SAS Institute).

## Results

### Smoking Prevalence, SHS Exposure, and Indoor Air Quality Before Policy Implementation

Telephone survey results suggested that residents across both building types were similar in demographic and smoking-related characteristics. Among 1030 residents living in NYCHA and Section 8 buildings who responded to the survey, 381 of 1008 respondents (37.8%) reported having children younger than 18 years, 222 of 980 (22.7%) had less than a high school educational level, 157 of 1013 (15.5%) reported current (past 30-day) smoking, 106 of 1019 (10.4%) reported living with an individual who smokes, and 741 of 1008 (73.5%) indicated they had a voluntary no-smoking policy in their apartment (Table 1). Despite comparable smoking prevalence, a higher proportion of NYCHA residents than Section 8 residents reported seeing others smoking in common areas (409 of 529 [77.3%] vs 245 of 441 [55.6%]; *P* < .001) and smelling cigarette smoke coming into their apartment (346 of 555 [62.3%] vs 266 of 465 [57.2%]; *P* = .10).

**Table 1.** Characteristics of Residents Living in 2 High-Rise Subsidized NYC Housing Settings Before the Implementation of the Smoke-Free Housing Policy in 2018

Characteristic	Residents, No. (%)^a^			*P* value^b^
	Overall	Residents in 10 NYC Housing Authority buildings	Residents in 11 Section 8 buildings	
Baseline telephone survey, No.	1030	559	471	NA
Demographic characteristics				
Children in home	381 (37.8)	206 (37.4)	175 (38.3)	.77
Educational level of at least high school graduate	758 (77.5)	409 (75.7)	349 (79.3)	.18
Spanish language	286 (28.4)	158 (28.5)	128 (28.3)	.85
Self-reported cigarette smoking behaviors				
Current smoking	157 (15.5)	87 (15.7)	70 (15.2)	.83
Individuals who smoke in the home	106 (10.4)	55 (9.9)	51 (11.0)	.57
Smoke-free policy in the home	741 (73.5)	411 (74.6)	330 (72.2)	.37
Self-reported SHS incursions				
Saw smoke in common areas	654 (67.4)	409 (77.3)	245 (55.6)	<.001
Smelled cigarette smoke in past year	612 (60.0)	346 (62.3)	266 (57.2)	.10
Households enrolled in longitudinal air monitoring, No.	263	153	110	NA
Race/ethnicity				
Non-Hispanic Black	105 (39.4)	61 (38.9)	44 (40.0)	.37
Hispanic or Latino	137 (51.5)	82 (52.2)	55 (50.0)	
Asian	2 (0.6)	2 (1.3)	0	
Non-Hispanic White	3 (1.1)	2 (1.3)	1 (0.9)	
American Indian/Alaska Native	2 (0.7)	1 (0.6)	1 (0.9)	
>1 Race/ethnicity	10 (4.1)	3 (1.9)	7 (6.4)	
Other	8 (2.7)	6 (3.8)	2 (1.8)	
Primary language spoken				
English	195 (72.3)	112 (71.3)	83 (73.4)	.75
Spanish	75 (27.7)	45 (28.7)	30 (26.6)	
No. of adults in the home				
1	109 (41.4)	63 (40.9)	46 (40.0)	.30
2	89 (33.0)	46 (29.9)	43 (37.4)	
≥3	71 (25.6)	45 (29.2)	26 (22.6)	
No. of children in the home				
0	154 (57.8)	87 (56.5)	67 (58.3)	.84
1	52 (19.2)	30 (19.5)	22 (19.1)	
2	38 (14.2)	21 (13.6)	17 (14.8)	
≥3	25 (8.8)	16 (10.4)	9 (7.8)	
Self-reported SHS incursions				
Smelled cigarette smoke in past 7 d	156 (58.2)	97 (63.4)	59 (53.6)	.047
Smelled marijuana smoke in past 7 d	127 (57.7)	59 (54.6)	68 (60.7)	.36
Saw cigarette use in common areas in past 7 d	141 (52.4)	93 (60.4)	48 (41.7)	.002
Saw e-cigarette use in common areas in past 7 d	20 (7.7)	13 (8.8)	7 (6.1)	.42
Factors affecting PM <2.5 μm in past 7 d				
Open windows	220 (81.6)	128 (83.1)	92 (80.0)	.95
Air conditioner use	176 (64.1)	113 (73.4)	63 (54.8)	.047
Incense, air freshener, or candles	170 (63.6)	94 (61.0)	76 (66.1)	.91
Kitchen stove use	252 (94.1)	141 (91.6)	111 (96.5)	.18
Humidifier use	21 (7.7)	13 (8.4)	8 (7.0)	.35
Vacuumed or swept	231 (85.7)	134 (87.0)	97 (84.4)	.62

Abbreviations: NA, not applicable; NYC, New York City; PM, particulate matter; SHS, secondhand smoke.

^a^ Percentages for each survey question may vary slightly owing to missing values, ranging from <1% to 5% missing.

^b^ *P* values were calculated using χ^2^ tests for independence.

Across building types, demographic and household composition characteristics of the 263 respondents from nonsmoking households participating in the longitudinal air-monitoring study were also similar, as was the frequency of other household behaviors likely associated with PM_2.5_ levels. Although residents in both building types reported comparable levels of smelling marijuana smoke and witnessing e-cigarette use in common areas, a higher proportion of NYCHA residents than Section 8 residents reported cigarette SHS exposure (smelled cigarette smoke in past 7 days: 97 of 153 [63.4%] vs 59 of 110 [53.6%]) (Table 1).

In the summer months immediately before policy implementation, measures of air nicotine and PM_2.5_ were higher in NYCHA buildings than in Section 8 buildings (Table 2). Nicotine was detectable in nearly all NYCHA and Section 8 stairwells (19 of 20 [95.0%] vs 15 of 19 [78.9%]; *P* = .19). In hallways, nicotine was detectable more frequently in NYCHA buildings than in Section 8 buildings (17 of 19 [89.5%] vs 14 of 23 [60.9%]; *P* = .004). Nicotine concentrations were lower inside apartments compared with in stairwells and hallways overall but were more frequently detected in NYCHA apartments than in Section 8 apartments (20 of 153 [13.1%] vs 6 of 110 [5.5%]; *P* = .04). Mean (SD) PM_2.5_ levels were also higher in NYCHA apartments than in Section 8 apartments (22.1 [10.8] μg/m^3^ vs 19.5 [10.5] μg/m^3^; *P* = .06), and a higher proportion of NYCHA apartments than Section 8 apartments had PM_2.5_ levels exceeding the Environmental Protection Agency annual health standard of 12 μg/m^3^ (123 of 137 [89.8%] vs 78 of 105 [74.3%]; *P* = .001). Before policy implementation, there was no significant difference in the mean (SD) number of cigarette butts in NYCHA and Section 8 stairwells (9.85 [7.76] vs 10.32 [7.98] per stairwell; *P* = .92), but mean (SD) counts of cigarette butts were higher in NYCHA hallways than in Section 8 hallways (4.35 [5.20] vs 0.95 [1.08] per hallway; *P* = .12).

**Table 2.** Air Quality Measures Across 2 Low-Income Subsidized Housing Settings in NYC Before vs 12 Months After SFH Policy Implementation^a^

Variable	Before SFH policy		12 mo After SFH policy	
	NYCHA	Section 8	NYCHA	Section 8
**Airborne nicotine**				
Stairwells				
No.	20	19	20	21
Level, geometric mean (SD), μg/m^3^	0.43 (0.11)	0.24 (0.10)	0.32 (0.05)	0.17 (0.06)
Proportion above LOD, %	95.0	78.9	100	85.7
Hallways				
No.	19	23	25	23
Level, geometric mean (SD), μg/m^3^	0.45 (0.16)	0.09 (0.03)	0.21 (0.06)	0.08 (0.02)
Proportion above LOD, %	89.5	60.9	84.0	73.9
Nonsmoking apartments				
No.	153	110	124	98
Level, geometric mean (SD), μg/m^3^	0.023 (0.002)	0.019 (0.001)	0.024 (0.002)	0.023 (0.002)
Proportion above LOD, %	13.1	5.5	24.2	16.3
**Particulate matter <2.5 μm**				
Nonsmoking apartments				
No.	137	105	126	99
Level, mean (SD), μg/m^3^	22.10 (10.80)	19.48 (10.49)	20.78 (11.26)	16.71 (7.35)
Above EPA standard, %	89.8	74.3	80.9	77.8
**Cigarette butt count**				
Stairwells				
No.	10	11	10	11
Count, mean (SD)	9.85 (7.76)	10.32 (7.98)	8.85 (5.35)	4.13 (3.72)
Hallways				
No.	10	11	10	11
Count, mean (SD)	4.35 (5.20)	0.95 (1.08)	2.15 (1.73)	0.45 (0.61)

Abbreviations: EPA, Environmental Protection Agency; LOD, limit of detection; NYC, New York City; NYCHA, NYC Housing Authority; SFH, smoke-free housing.

^a^ Measures before the policy are from summer 2018, and measures after the policy are from summer 2019; all measures were taken from 10 NYCHA buildings and 11 Section 8 buildings.

### Changes in Indoor Air Quality 1 Year After Policy Implementation

In summer 2019, one year after policy implementation, air quality was measured again and field staff repeated systematic cigarette butt counts. In NYCHA common areas and in nonsmoking apartments, introduction of the SFH policy was not associated with differential reductions in air nicotine compared with similar settings in Section 8 buildings (Table 3). Overall, nicotine concentrations remained higher in NYCHA settings (Figure). In apartments and stairwells, temporal changes in air nicotine levels were similar between NYCHA and Section 8 buildings, and the magnitude of differential change between arms was negligible and not statistically significant (apartment DID: –0.04 μg/m^3^ [95% CI, −0.24 to 0.15 μg/m^3^], *P* = .67; stairwell DID: 0.03 μg/m^3^ [95% CI, −0.99 to 1.06 μg/m^3^], *P* = .95). In hallways, decreases were larger in NYCHA buildings for air nicotine (DID, –0.43 μg/m^3^; 95% CI, −1.26 to 0.40 μg/m^3^; *P* = .30) and cigarette butts (DID, –1.70 μg/m^3^; 95% CI, −4.30 to 0.90 μg/m^3^; *P* = .19), but the differences were not statistically significant. Before adjustment for ambient air quality, indoor PM_2.5_ levels were decreased in both NYCHA and Section 8 apartments, but decreases were larger in Section 8 apartments (Table 2). After adjustment for ambient air quality, the difference in indoor PM_2.5_ levels in Section 8 apartments compared with NYCHA apartments was even larger. Variability in mean stairwell cigarette butt counts across buildings was not statistically significant, but reductions were larger for Section 8 buildings (DID, 5.18; 95% CI, −3.15 to 13.51; *P* = .21) (Table 3).

**Table 3.** Difference-in-Difference Model Results for Change in Air Quality Measures From Before to 12 Months After Smoke-Free Housing Policy Implementation Across 2 Low-Income Subsidized Housing Settings in NYC

Effect	Difference in particle concentrations before policy to 12 mo after policy, mean (95% CI), μg/m^3^	Difference-in-difference estimate (95% CI)	*P* value
**Airborne nicotine concentration**^**a**^			
Stairwells			
NYCHA	−0.29 (−1.03 to 0.45)	0.03 (−0.99 to 1.06)	.95
Section 8	−0.32 (−1.04 to 0.39)		
Hallways			
NYCHA	−0.60 (−1.19 to 0.01)	−0.43 (−1.26 to 0.40)	.30
Section 8	−0.16 (−0.74 to 0.41)		
Nonsmoking apartments			
NYCHA	0.12 (−0.01 to 0.24)	−0.04 (−0.24 to 0.15)	.67
Section 8	0.16 (0.01 to 0.31)		
**Particulate matter <2.5 μm (unadjusted)**			
Nonsmoking apartments			
NYCHA	−1.44 (−3.73 to 0.84)	1.36 (−2.10 to 4.81)	.44
Section 8	−2.80 (−5.39 to −0.21)		
**Particulate matter <2.5 μm (adjusted)**^**b**^			
Nonsmoking apartments			
NYCHA	0.91 (−1.33 to 3.15)	3.92 (0.59 to 7.25)	.02
Section 8	−3.02 (−5.49 to −0.55)		
**Cigarette butt count**			
Stairwells			
NYCHA	−1.00 (−7.03 to 5.03)	5.18 (−3.15 to 13.51)	.21
Section 8	−6.18 (−11.93 to −0.44)		
Hallways			
NYCHA	−2.20 (−4.09 to −0.32)	−1.70 (−4.30 to 0.90)	.19
Section 8	−0.50 (−2.30 to 1.30)		

Abbreviation: NYC, New York City; NYCHA, NYC Housing Authority.

^a^ Outcome, natural log of air nicotine concentration, or ln(nicotine).

^b^ Adjusted for ambient outdoor levels of particulate matter less than 2.5 μm.

**Figure.**
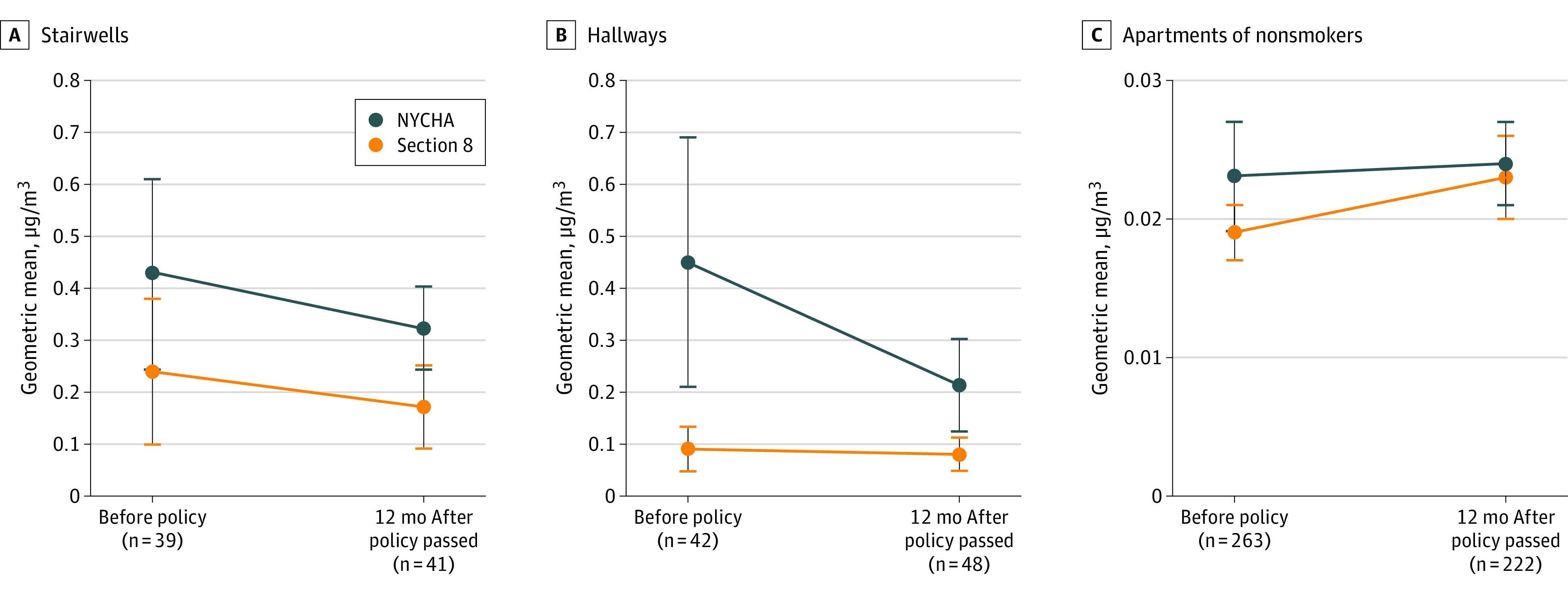
Airborne Nicotine Levels in New York City Housing Authority (NYCHA) and Section 8 Buildings Before and 12 Months After Passage of the Smoke-Free Housing Policy in 2018 Section 8 buildings are private-sector buildings in which households may receive vouchers to subsidize apartment rental costs. Whiskers indicate 95% CIs.

## Discussion

This study evaluated whether a federal policy to ban smoking in public housing authority buildings was associated with reductions in air nicotine and PM_2.5_ levels 1 year after the policy went into effect with use of a natural experiment design and extensive monitoring in a sample of NYC public housing and Section 8 high-rise buildings. We found no differential change in air nicotine levels in NYCHA apartments or stairwells compared with Section 8 buildings. Although modest reductions in air nicotine levels were observed in NYCHA vs Section 8 hallways over time, a finding corroborated by fewer observed cigarette butts, we cannot rule out the possibility of chance in these modest changes. Continued monitoring may confirm whether NYCHA residents are smoking less frequently in visible common areas, such as hallways, owing to the SFH policy and whether they continue to smoke in stairwells and inside homes, where neighbors may be less likely to notice or complain.

The findings of this study are consistent with those of other studies examining the association of SFH policies with SHS exposure.^15,16,18^ One recent study^18^ examined the consequences of the HUD SFH policy 1 year after policy implementation in only the common areas of 6 public housing buildings in Norfolk, Virginia. The authors identified modest decreases in PM_2.5_ and airborne nicotine 1 month immediately after policy implementation, but levels increased 12 months after policy implementation, surpassing even prior-year levels. Another study^15^ assessed the association of a voluntary SFH policy with SHS exposure in Philadelphia public housing in a noncontrolled air quality study of approximately 30 nonsmoking households and 30 common areas at 3 time points: 1 and 2 years before and 9 months after policy implementation. Although airborne nicotine had decreased significantly in public areas (–0.19 μg/m^3^) 9 months after policy implementation, the number of locations with detectable nicotine was similar across all 3 time points. One of the earliest studies^16^ published on SFH policy consequences examined week-long airborne nicotine and PM_2.5_ samples across common areas of 10 Boston Housing Authority buildings affected by a 2012 municipal SFH policy and 6 Cambridge Housing Authority buildings with no SFH policy. In contrast with other studies, these authors found that Boston SFH policy implementation was associated with greater decreases in PM_2.5_ and airborne nicotine concentration 1 year after the policy implementation compared with housing that had no policy, although the association was not equal at all sites.^16^ To our knowledge, evidence of persistently reduced SHS over time has not been published.

Variation across studies may reflect differences in how policies are implemented or may be an artifact of measurement protocols. For example, in Boston, violators of the SFH policy were subject to a fine and possible eviction,^33^ while Philadelphia’s policy was voluntary.^34^ In NYCHA buildings, residents who repeatedly violate the policy are subject to possible eviction. Because this is not desirable and eviction is mostly intended as a deterrent, the NYCHA has not evicted anyone on this basis to date, to our knowledge. Variation in evaluation protocol is also plausible. Whereas the filters and testing laboratory used in the current study were the same as those used in the Virginia and Philadelphia studies, the Boston study used a different laboratory and filters. A strength of this study is that it included a substantially larger sample of nonsmoking households (263) and indoor common areas (more than 90) than prior studies and included a concurrent comparison arm.

To reduce SHS exposures and disparities in tobacco-related outcomes in public housing and other multiunit housing settings, a greater understanding of challenges to policy compliance and enforcement is needed. Prior qualitative analyses identified a range of barriers to implementing SFH policies.^35,36^ These include a sentiment among many residents that they have a right to privacy and autonomy in their homes (counterbalancing overall support for the policy), a lack of tobacco cessation treatment support, and frustration that other building violations, such as marijuana use, are not being addressed. A study by Hernández and colleagues^35^ suggests that compliance is negatively affected when social contracts regarding the mutual responsibilities that residents and building management have to each other are not upheld. Strategies for improving SFH policy effectiveness may include providing more support for smokers to quit, greater community engagement, investments to improve environmental and structural health of buildings, and integrating the SFH agenda into a wider healthy-homes policy agenda. Continued evaluation of the implementation process and assessment of consequences for health outcomes is critical to optimize both. Given that small changes in annual SHS exposure (ie, 10 μg/m^3^) are potentially associated with longer-term health outcomes, even modest decreases in nicotine levels may have demonstrable consequences for health over time.^31,34,37,38,39^ As public housing authorities promote smoke-free living conditions, longitudinal studies are needed to monitor the association of SFH policies with air quality and health outcomes.

### Limitations

This study has limitations. First, buildings selected for monitoring were not randomly chosen, which may limit generalizability of findings to all NYC public housing buildings. However, building criteria were determined a priori, and buildings were purposefully selected from a small list in consultation with NYCHA managers, taking into account factors likely to affect recruitment (eg, events affecting resident trust) or air quality (eg, nearby construction). In addition, similarity in resident characteristics, smoking prevalence, e-cigarette use, other household behaviors associated with PM_2.5_ levels, and the buildings’ structural characteristics between final samples of NYCHA and Section 8 buildings, as well as high response rates in both groups, suggests high internal validity and applicability to other buildings. Second, air quality monitoring equipment is subject to variability. To address this, PM_2.5_ monitors were calibrated before each sampling wave using cigarette smoke. Passive nicotine monitors have been extensively validated in previous studies,^15,40,41,42^ although a lower limit of detection would have permitted more detailed evaluation of SHS exposure in nonsmoking households. Third, the DID analysis included only 1 round of prepolicy measurements, limiting the ability to verify the parallel-trends assumption in comparison groups before policy intervention.

## Conclusions

In this cohort study of air quality, HUD SFH policy was not associated with reduced indoor air nicotine levels. These findings suggest that in its first year, the HUD-issued SFH policy was not associated with SHS levels in the largest housing authority in the US. Additional efforts to support policy enforcement, provide cessation services, and raise awareness of health benefits of reduced SHS exposure are needed.

## Notes

Supplement. **eFigure.** Flow Diagram for Longitudinal Air Monitoring by Study Arm

## References

[1] California Environmental Protection Agency Proposed Identification of Environmental Tobacco Smoke as a Toxic Air Contaminant. California Environmental Protection Agency; 2005.

[2] US Department of Health and Human Services The Health Consequences of Involuntary Exposure to Tobacco Smoke: A Report of the Surgeon General. Office of the Surgeon General; 2006.

[3] US Department of Health and Human Services The Health Consequences of Smoking—50 Years of Progress: A Report of the Surgeon General. Office of the Surgeon General; 2014.

[4] Tsai J, Homa DM, Gentzke AS, Exposure to secondhand smoke among nonsmokers—United States, 1988-2014. MMWR Morb Mortal Wkly Rep. 2018;67(48):1342-1346. doi:10.15585/mmwr.mm6748a3 30521502 PMC6329485

[5] Chen CI, Burton T, Baker CL, Mastey V, Mannino D Recent trends in exposure to secondhand smoke in the United States population. BMC Public Health. 2010;10:359. doi:10.1186/1471-2458-10-359 20573192 PMC2912805

[6] Helms VE, King BA, Ashley PJ Cigarette smoking and adverse health outcomes among adults receiving federal housing assistance. Prev Med. 2017;99:171-177. doi:10.1016/j.ypmed.2017.02.001 28192095 PMC5508864

[7] Jamal A, Homa DM, O’Connor E, Current cigarette smoking among adults—United States, 2005-2014. MMWR Morb Mortal Wkly Rep. 2015;64(44):1233-1240. doi:10.15585/mmwr.mm6444a2 26562061

[8] King BA, Peck RM, Babb SD National and state cost savings associated with prohibiting smoking in subsidized and public housing in the United States. Prev Chronic Dis. 2014;11:E171. doi:10.5888/pcd11.140222 25275808 PMC4184089

[9] Lopez PM, Islam N, Feinberg A, A place-based community health worker program: feasibility and early outcomes, New York City, 2015. Am J Prev Med. 2017;52(3)(suppl 3):S284-S289. doi:10.1016/j.amepre.2016.08.034 28215382 PMC5656273

[10] King BA, Cummings KM, Mahoney MC, Juster HR, Hyland AJ Multiunit housing residents’ experiences and attitudes toward smoke-free policies. Nicotine Tob Res. 2010;12(6):598-605. doi:10.1093/ntr/ntq053 20395360 PMC3436441

[11] Kraev TA, Adamkiewicz G, Hammond SK, Spengler JD Indoor concentrations of nicotine in low-income, multi-unit housing: associations with smoking behaviours and housing characteristics. Tob Control. 2009;18(6):438-444. doi:10.1136/tc.2009.029728 19679890 PMC5624306

[12] Nguyen KH, Gomez Y, Homa DM, King BA Tobacco use, secondhand smoke, and smoke-free home rules in multiunit housing. Am J Prev Med. 2016;51(5):682-692. doi:10.1016/j.amepre.2016.05.009 27423656 PMC5821129

[13] Homa DM, Neff LJ, King BA, ; Centers for Disease Control and Prevention Vital signs: disparities in nonsmokers’ exposure to secondhand smoke—United States, 1999-2012. MMWR Morb Mortal Wkly Rep. 2015;64(4):103-108. 25654612 PMC4584848

[14] US Department of Housing and Urban Development *Instituting Smoke-Free Public Housing: A Proposed Rule by the Housing and Urban Development Department* Accessed October 6, 2020. https://portal.hud.gov/hudportal/documents/huddoc?id=smoke-freepublichousing.pdf

[15] Klassen AC, Lee NL, Pankiewicz A, Secondhand smoke exposure and smoke-free policy in Philadelphia public housing. Tob Regul Sci. 2017;3(2):192-203. doi:10.18001/TRS.3.2.7 28944277 PMC5609462

[16] MacNaughton P, Adamkiewicz G, Arku RE, Vallarino J, Levy DE The impact of a smoke-free policy on environmental tobacco smoke exposure in public housing developments. Sci Total Environ. 2016;557-558:676-680. doi:10.1016/j.scitotenv.2016.03.110 27037889 PMC4856038

[17] Phillips K, Bentley MC, Howard DA, Alván G Assessment of environmental tobacco smoke and respirable suspended particle exposures for nonsmokers in Prague using personal monitoring. Int Arch Occup Environ Health. 1998;71(6):379-390. doi:10.1007/s004200050296 9766911

[18] Plunk AD, Rees VW, Jeng A, Wray JA, Grucza RA Increases in secondhand smoke after going smoke free: an assessment of the impact of a mandated smoke-free housing policy. Nicotine Tob Res. 2020;ntaa040. doi:10.1093/ntr/ntaa040 32080738 PMC7899270

[19] Russo ET, Hulse TE, Adamkiewicz G, Comparison of indoor air quality in smoke-permitted and smoke-free multiunit housing: findings from the Boston Housing Authority. Nicotine Tob Res. 2015;17(3):316-322. doi:10.1093/ntr/ntu146 25156526 PMC4837992

[20] Hollar TL, Cook N, Quinn D, Phillips T, DeLucca M Smoke-free multi-unit housing policies show promise in reducing secondhand smoke exposure among racially and ethnically diverse, low-income seniors. J Immigr Minor Health. 2017;19(6):1281-1289. doi:10.1007/s10903-016-0430-2 27189486

[21] US Centers for Disease Control and Prevention Transparent reporting of evaluations with nonrandomized designs. Updated September 26, 2018. Accessed September 29, 2020. https://www.cdc.gov/trendstatement/

[22] Cardozo RA, Feinberg A, Tovar A, A protocol for measuring the impact of a smoke-free housing policy on indoor tobacco smoke exposure. BMC Public Health. 2019;19(1):666. doi:10.1186/s12889-019-7043-3 31146711 PMC6543633

[23] Anastasiou E, Feinberg A, Tovar A, Secondhand smoke exposure in public and private high-rise multiunit housing serving low-income residents in New York City prior to federal smoking ban in public housing, 2018. Sci Total Environ. 2020;704:135322. doi:10.1016/j.scitotenv.2019.135322 31787288 PMC6939143

[24] Avila-Tang E, Elf JL, Cummings KM, Assessing secondhand smoke exposure with reported measures. Tob Control. 2013;22(3):156-163. doi:10.1136/tobaccocontrol-2011-050296 22949496 PMC3639349

[25] Benowitz NL Cotinine as a biomarker of environmental tobacco smoke exposure. Epidemiol Rev. 1996;18(2):188-204. doi:10.1093/oxfordjournals.epirev.a017925 9021312

[26] Rosen LJ, Myers V, Winickoff JP, Kott J Effectiveness of interventions to reduce tobacco smoke pollution in homes: a systematic review and meta-analysis. Int J Environ Res Public Health. 2015;12(12):16043-16059. doi:10.3390/ijerph121215038 26694440 PMC4690974

[27] Apelberg BJ, Hepp LM, Avila-Tang E, Environmental monitoring of secondhand smoke exposure. Tob Control. 2013;22(3):147-155. doi:10.1136/tobaccocontrol-2011-050301 22949497 PMC3639351

[28] Hammond SK, Leaderer BP A diffusion monitor to measure exposure to passive smoking. Environ Sci Technol. 1987;21(5):494-497. doi:10.1021/es00159a012 22296139

[29] Johns Hopkins Bloomberg School of Public Health Secondhand smoke monitoring: analysis. Accessed September 29, 2020. http://www.shsmonitoring.org/analysis/lab/

[30] Palmes ED, Burton RM Jr, Ravishankar K, Solomon JJ A simple mathematical model for diffusional sampler operation. Am Ind Hyg Assoc J. 1986;47(7):418-420. doi:10.1080/15298668691389973 3751889

[31] National ambient air quality standards for particulate matter. *Fed Regist.* 2013;78(10):3086-3287. 40 CFR Parts 50, 51, 52, 53, and 58. Accessed October 6, 2020. https://www.gpo.gov/fdsys/pkg/FR-2013-01-15/pdf/2012-30946.pdf

[32] New York City Department of Health and Mental Hygiene New York City Community Air Survey. 2017 Accessed September 29, 2020. https://www1.nyc.gov/site/doh/data/data-publications/air-quality-nyc-community-air-survey.page

[33] Anthony J, Goldman R, Rees VW, Qualitative assessment of smoke-free policy implementation in low-income housing: enhancing resident compliance. Am J Health Promot. 2019;33(1):107-117. doi:10.1177/0890117118776090 29772910 PMC10623451

[34] Puett RC, Hart JE, Yanosky JD, Chronic fine and coarse particulate exposure, mortality, and coronary heart disease in the Nurses’ Health Study. Environ Health Perspect. 2009;117(11):1697-1701. doi:10.1289/ehp.0900572 20049120 PMC2801178

[35] Hernández D, Swope CB, Azuogu C, Siegel E, Giovenco DP “If I pay rent, I’m gonna smoke”: insights on the social contract of smokefree housing policy in affordable housing settings. Health Place. 2019;56:106-117. doi:10.1016/j.healthplace.2019.01.007 30716667 PMC7146084

[36] Jiang N, Thorpe L, Kaplan S, Shelley D Perceptions about the federally mandated smoke-free housing policy among residents living in public housing in New York City. Int J Environ Res Public Health. 2018;15(10):15. doi:10.3390/ijerph15102062 30241291 PMC6210957

[37] Eftim SE, Samet JM, Janes H, McDermott A, Dominici F Fine particulate matter and mortality: a comparison of the six cities and American Cancer Society cohorts with a Medicare cohort. Epidemiology. 2008;19(2):209-216. doi:10.1097/EDE.0b013e3181632c09 18223484

[38] Lepeule J, Laden F, Dockery D, Schwartz J Chronic exposure to fine particles and mortality: an extended follow-up of the Harvard Six Cities study from 1974 to 2009. Environ Health Perspect. 2012;120(7):965-970. doi:10.1289/ehp.1104660 22456598 PMC3404667

[39] Pope CA III, Burnett RT, Thun MJ, Lung cancer, cardiopulmonary mortality, and long-term exposure to fine particulate air pollution. JAMA. 2002;287(9):1132-1141. doi:10.1001/jama.287.9.1132 11879110 PMC4037163

[40] Kim S, Wipfli H, Navas-Acien A, ; FAMRI Homes Study Investigators Determinants of hair nicotine concentrations in nonsmoking women and children: a multicountry study of secondhand smoke exposure in homes. Cancer Epidemiol Biomarkers Prev. 2009;18(12):3407-3414. doi:10.1158/1055-9965.EPI-09-0337 19959689

[41] Torrey CM, Moon KA, Williams DA, Waterpipe cafes in Baltimore, Maryland: carbon monoxide, particulate matter, and nicotine exposure. J Expo Sci Environ Epidemiol. 2015;25(4):405-410. doi:10.1038/jes.2014.19 24736103 PMC4333110

[42] Navas-Acien A, Peruga A, Breysse P, Secondhand tobacco smoke in public places in Latin America, 2002-2003. JAMA. 2004;291(22):2741-2745. doi:10.1001/jama.291.22.2741 15187056

