# Supplementary material for: Evaluation of Secondhand Smoke Exposure in New York City Public Housing After Implementation of the 2018 Federal Smoke-Free Housing Policy
Source: JAMA Netw Open. 2020 Nov 5;3(11):e2024385. doi: 10.1001/jamanetworkopen.2020.24385 (PMC7645700; doi:10.1001/jamanetworkopen.2020.24385)
Supplement: Supplement. — eFigure. Flow Diagram for Longitudinal Air Monitoring by Study Arm [file jamanetwopen-e2024385-s001.pdf]

Supplementary Online Content

Thorpe LE, Anastasiou E, Wyka K, et al. Evaluation of secondhand smoke exposure in New York City public housing after implementation of the 2018 federal smoke-free housing policy. *JAMA Netw Open*. 2020;3(11):e2024385. doi:10.1001/jamanetworkopen.2020.24385

eFigure. Flow Diagram for Longitudinal Air Monitoring by Study Arm

This supplementary material has been provided by the authors to give readers additional information about their work.

© 2020 Thorpe LE et al. *JAMA Network Open*.

eFigure. Flow Diagram for Longitudinal Air Monitoring by Study Arm

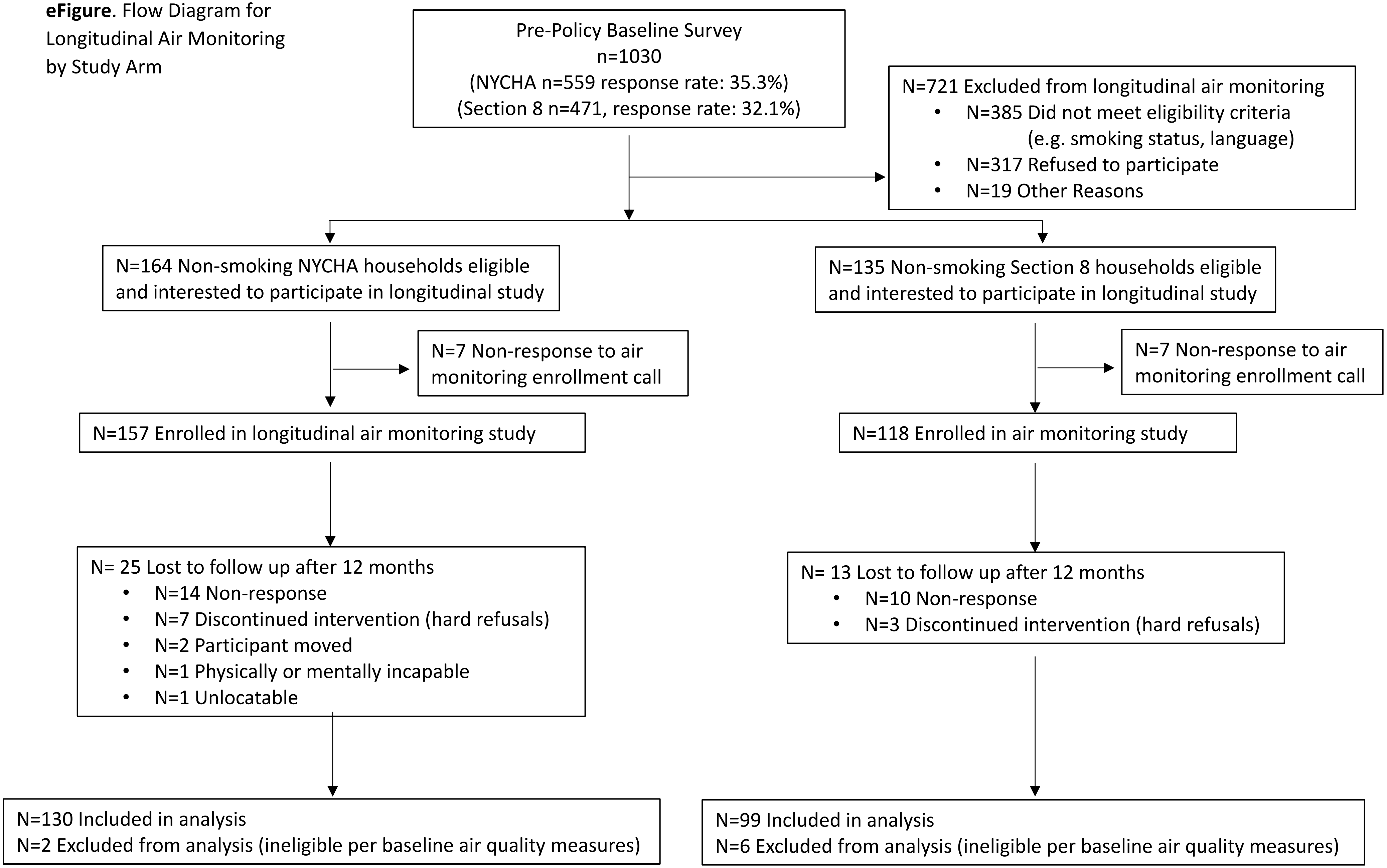

© 2020 Thorpe LE et al. *JAMA Network Open*.
